# Pre-Exposure Prophylaxis with Hydroxychloroquine Does Not Prevent COVID-19 nor Virus Related Venous Thromboembolism

**DOI:** 10.3390/v13102052

**Published:** 2021-10-13

**Authors:** Alessandro Perrella, Valentina Orlando, Ugo Trama, Francesca F. Bernardi, Enrica Menditto, Enrico Coscioni

**Affiliations:** 1 Hospital Health Direction, Infectious Disease Unit, Hospital A. Cardarelli, 80131 Naples, Italy; 2 CIRFF, Center of Pharmacoeconomics, University of Naples Federico II, 80131 Naples, Italy; valentina.orlando@unina.it (V.O.); enrica.menditto@unina.it (E.M.); 3 Regional Direction for Health Management, Pharmaceutical Unit, 80131 Naples, Italy; ugo.trama@regione.campania.it (U.T.); bernardi.francesca.futura@gmail.com (F.F.B.); 4 Division of Cardiac Surgery, AOU San Giovanni di Dio e Ruggi d’Aragona, 84131 Salerno, Italy; coscionienrico@gmail.com

**Keywords:** COVID-19, venous thromboembolism, SARS-CoV-2, hydroxychloroquine

## Abstract

Different and several public health strategies have been planned to reduce transmission of pandemic due to SARS-CoV-2 since it started. None drugs have been confirmed as able to prevent viral transmission. Hydroxychloroquine with its immunomodulatory properties has been proposed as potential anti-viral drug in particular for prevention once viral exposure has been happen or in first phases of infection. Furthermore, in several immunological systemic disease hydroxychloroquine was able to reduce the number of thrombotic complications. So, because COVID-19 was associated to immunological imbalance and to thrombotic complications, we retrospectively analyzed the rate of infection in those patients being under treatment with this drug during COVID-19 epidemic outbreak from 8 March until 28 April in particular comparing those with pre-exposure to this treatment and those that were not taking this medication before SARS-CoV-2 viral infections.

## 1. Introduction

Since its first description in December 2019, SARS-CoV-2, causing Coronavirus Disease (COVID-19), has rapidly spread worldwide, requiring a multidisciplinary approach, need for treatments, as well as preventive strategies to contain the disease spread [1]. Currently, global public health strategies to reduce transmission are based on rapid identification of cases, isolation, contact tracing, and quarantine of those exposed and infected. In some cases, forecasting analysis strategies have been also proposed and used to understand and therefore modify the trend of the outbreak [2]. Once a person has been exposed, observation and quarantine during a 14-day incubation period are the current standards of care. Whereas symptomatic subjects may be hospitalized according to the severity of the disease presentation, while asymptomatic are in active surveillance. Now, even if several treatment schedules have been proposed, no medication has been shown to prevent or completely treat the SARS-CoV-2 transmission [3]. Among the proposed drugs, hydroxychloroquine alone or coupled with azithromycin has been suggested to be potentially useful as an antiviral and anti-inflammatory [4]. Particularly, it has been demonstrated that hydroxychloroquine is thought to impair the terminal glycosylation of the angiotensin-converting–enzyme 2 (ACE2) receptor, which is the binding site for the envelope spike glycoprotein and has been shown to inhibit endolysosome function with an increased activity compared to chloroquine [4]. Furthermore, the common use of hydroxychloroquine in chronic systemic diseases, such as systemic erythematosus lupus, is also associated with a decrease in thrombotic complications [5]. Therefore, since COVID-19 is associated with an increased rate of thrombotic complications, it could be speculated that the use of hydroxychloroquine may also eventfully reduce the rate of thrombotic complications in those who have COVID-19 despite the use of a chloroquine derived drug. Yet, most of the currently published papers on this medication and its impact on COVID-19 have focused on hospitalized patients and recently as post-expositional prophylaxis to prevent disease soon after a high-risk exposure in close contacts of infected patients, while data on the useful effect of a pre-exposure use of hydroxychloroquine in a large cohort of subjects at risk of developing COVID-19 are lacking in the literature. According to previous evidence on the possible role of hydroxychloroquine, we aimed to very if this drug could potentially have a role as pre-exposure prophylaxis, such as in HIV, to prevent COVID-19 in those subjects who are under treatment for other illness, such as autoimmune disease.

## 2. Methods

Since first report of possible in vitro activity of hydroxychloroquine [4], in Campania region, we evaluated the rate of infection in those patients being under treatment with this drug during COVID-19 epidemic outbreak from 8 March until 28 April. These patients would have been considered as in a pre–exposure prophylaxis (PrEP) as for HIV [6] and potentially protected according to the possible effect of hydroxychloroquine in vitro [4].

### Target Population and Data Source

A retrospective drug-utilization study was carried out using routinely collected information from healthcare databases in Campania [7,8,9] The Campania Region Database (CaReDB) includes information on patient demographics and the electronic records of outpatient pharmacy dispensing for ~6 million residents, comprising a well-defined population in Italy (~10% of the population of Italy). CaReDB is complete and includes data that has been validated in previous drug-utilization studies on COVID-19 [10,11,12]. The characteristics of CaReDB are described in Supplementary Table S1.

People who had been dispensed medication “Hydroxychloroquine” according to CaReDB from 1 October until 31 December 2019 were included in the study cohort. From regional surveillance system data, we obtained the information of patients with con-firmed COVID-19 from the beginning of the epidemic (26 February 2020) to 28 April 2020 who were linked to the population identified in CaReDB. For the purposes of our investigation, the study population diagnosed with SARS-CoV-2 infection on or before the date of analysis was referred to as the ‘COVID-19 group’ (C19G). The remaining individuals were used as a comparator group in the analysis and were referred to as the ‘general population group’ (GPG).

## 3. Cases and Controls

The date of SARS-CoV-2 infection diagnosis was considered as the index date and patients were extracted from the COVID-19 registry until 28 April 2020. A total of 3519 users positive to SARS-CoV-2 were identified included into the study as cases.

For each case, up to five controls were randomly selected from the target population to be matched for gender, age at index date and municipality of residence. The density incidence approach was used for selecting controls since patients who had a confirmed diagnosis of SARS-CoV-2 infection were eligible as potential controls until they became cases, and all matches had to be at risk of SARS-CoV-2 infection.

## 4. Statistical Methods

Crude and age-adjusted prevalence rates were calculated. Differences in the prevalence between the C19G and GPG are expressed as percentages adjusted for sex and age. Standardization was performed using a direct method whereby the Italian population up to 1 January 2019 was used as the standard population (available on the Demo Istat website http://demo.istat.it/ accessed on 15 May 2021).

$$\mathrm{Directly standardized rate}\;=\;\frac{\sum_{i=1}^{m}w_{i}\cdot T_{i}}{\sum_{i=1}^{m}w_{i}}\cdot k$$

where (*T_i_*  =  *n_i_*/*n*)  =  rate in stratum ‘*i*’ of the study population; *n_i_*  =  number of cases in stratum ‘*i*’ of the study population; *N*  =  size of the study population in stratum ‘*i*’; *w_i_*  =  size of stratum ‘*i*’ of the reference population; *m*  =  number of considered strata; *k*  =  multiplicative constant.

The age-adjusted RRs and 95% CIs were computed using standard methods. Data management was performed with SQL server v2018 (Microsoft, Redmond, WA, USA). Analyses were carried out with SPSS v17.1 (IBM, Armonk, NY, USA). Univariate and multivariate logistic regression models were performed among the whole case/control cohort, proving if the hydroxychloroquine use affects the risk of contracting or not SARS-CoV-2 infection.

At the beginning of the COVID-19 epidemic, a surveillance system was implemented to collect the data of all cases identified by reverse transcription-polymerase chain reaction (RT-PCR) testing for SARS-CoV-2. These archives are linked together by a unique anonymous identifier that is encrypted to protect patient privacy. Furthermore, the main outcomes regarding respiratory performance for patients pre-treated or not with hydroxychloroquine were analyzed (i.e., ARDS, use of non-invasive ventilation, venous thromboembolism, overall death) in those infected with SARS-CoV-2 virus. Particularly, 24 patients age- and sex-matched were randomly selected as ratio 1:2 to compare the differences in terms of clinical outcome according to thromboembolism events between those with COVID-19 not being under and those undergoing hydroxychloroquine. Univariate and multivariate logistic regression models were performed among the whole case/control cohort, proving if the hydroxychloroquine use affects the risk of contracting or not SARS-CoV-2 infection.

## 5. Results

In this study, we evaluated 8811 patients who underwent hydroxychloroquine treatment for different clinical conditions, and we compared them with a control group of 17,514 subjects living in the Campania Region. Evaluating the incidence of SARS-CoV-2 of those patients, we found that only 12 patients underwent SARS-CoV-2 infection (0.14% of hydroxychloroquine total users). At a statistical analysis, we did not find any significant difference in terms of risk of development of SARS-CoV-2 infection between those being treatment with hydroxychloroquine vs. those without, particularly at Unadjusted OR (95% CI) 4.96 (2.48–9.92); Adjusted OR (95% CI) 5.802 (2.82–11.93) (Table 1, Table 2 and Table 3). Furthermore, no differences, in terms of SARS-CoV-2 related complications, such as ARDS, as far as venous thromboembolisms, were recorded regarding the outcome of patients with COVID-19 pre-treated or not with hydroxychloroquine, as reported in Table 4. Moreover, a statistically significant difference in hydroxychloroquine prevalence use was recorded, higher for the C19G than the GPG (0.36% vs. 0.17%; RR, 2.12) (Table 1, Table 2 and Table 3).

## 6. Discussion

During COVID-19 Pandemic outbreak, due to the high mortality and critical illness many treatments have been proposed and used to reduce mortality and hospital stay with related complications. Among these drugs, hydroxychloroquine during the first phase of the pandemic outbreak has been suggested as possible efficacy treatment schedule. Therefore, scientific community hypothesized that this drug could potentially be used as pre-exposure prophylaxis, to prevent COVID-19. One of the strategies to assess if a drug could be useful again a disease is to assess its possible role preventing the illness as already demonstrated for HIV [6] and other disease [13]. According to our results we found that receiving hydroxychloroquine treatment does not represent a protective factor in contracting COVID-19 compared to those not under therapy. Furthermore a subsequent analysis on data of inpatients with COVID-19 pre-treated with hydroxychloroquine did not modify main outcomes compared with those not pretreated, on the contrary they showed a more severe clinical condition requiring noninvasive ventilation, as reported in Table 4. From a clinical point of view data of un-successful pre-treatment to prevent COVID-19 and the evidence of a more severe disease is interesting, and it could underline inflammatory mechanisms not related to possible HC activity. The effectiveness of hydroxychloroquine in systemic disease and\or antiphospholipid syndrome has been assessed also regarding the reduction of thrombotic rate. Particularly the reduction was associated to the increase of the dosage of hydroxychloroquine [14,15]. Indeed, the rate of venous thromboembolism in our cohort did not differ between the group that performed pre-exposure with hy-droxychloroquine and the group not receiving HC during the disease. Finally, based on these results and according to other published papers [1] hydroxychloroquine does not seem to confer any advantage both pre-exposure prophylaxis too. From a clinical point of view, we also verified this trend when main clinical outcomes as the rate of ARDS as far as of VTE were analyzed. These evidences should be carefully considered not only to avoid possible adverse drug effects of this drug and to prevent in future to start heroic and hazardous treatments without any real data, even in presence of a pandemic. However, we also ha e to say that our findings are mainly related to the first wave of COVID-19 global spread. Indeed, a possible future strategy to assess a potential use of any drug as antiviral should consider to assess an analysis of its possible role in prevent infection spread in those subsets of patients undergoing treatment with the speculated drug on.

## Figures and Tables

**Table 1:** Baseline characteristics of the study population.

Hydroxychloroquine Use (N)			
	Yes	No	Overall
GPG	8799	5,789,374	5,798,173
C19G	12	3507	3519
Total	8811	5,792,881	5,801,692

**Table 2:** Differences in prevalence of hydroxychloroquine use between C19G and GPG.

ATC V	Name	Prevalence of Drug Use (%)			
		Unadjusted		Adjusted	
		C19G	GPG	C19G	GPG
P01BA02	Hydroxychloroquine	0.34	0.15	0.36	0.17

**Table 3:** Univariate and multivariate logistics regression of the risk of contracting COVID-19.

	Unadjusted OR (95% CI)	*p*-Value	Adjusted OR (95% CI)	*p*-Value
**Hydroxychloroquine** **(vs. no Hydroxychloroquine)**	4.96 (2.48–9.92)	<0.01	5.802 (2.82–11.93)	<0.01
**Gender**				
Male (vs. Female)	0.99 (0.92–1.07)	0.981	1.311 (0.89–1.91)	0.160
**Age groups**				
40–59 years (vs. 0–39 years)	0.99 (0.90–1.09)	0.964	0.895 (0.56–1.41)	0.633
60–79 years (vs. 0–39 years)	1.00 (0.90–1.10)	0.998	0.781 (0.47–1.29)	0.335
≥80 years (vs. 0–39 years)	1.03 (0.89–1.18)	0.674	0.940 (0.46–1.91)	0.865

**Table 4:** Clinical evolution in patients with COVID-19 with and without pre-medication with hydroxychloroquine.

	COVID 19 Treated without HC	COVID 19 with Premedication with HC before COVID-19	*p*-Value
Patients, *n* (%)	24	12	
ARDS	6	4	n.s.
Use of non-invasive ventilation	7	6	<0.01
Associated venous thromboembolism	1	1	n.s.
Death for any reason	1	0	n.s.

Table shows the clinical complications of inpatients COVID-19 according to HC pre-exposure. n.s. means: not significant.

## Data Availability

Permission use anonymized data to this study was granted to the researchers of ENTE. Requests for information on data access can be directed to Perrella A and Orlando V.

## Supplementary Materials

The following are available online at https://www.mdpi.com/article/10.3390/v13102052/s1, Table S1: Campania Region Database (CaReDB) character.
