# Supplementary material for: Pre-Exposure Prophylaxis with Hydroxychloroquine Does Not Prevent COVID-19 nor Virus Related Venous Thromboembolism
Source: Viruses. 2021 Oct 13;13(10):2052. doi: 10.3390/v13102052 (PMC8537634; doi:10.3390/v13102052)
Supplement: Supplementary file 1 [file viruses-13-02052-s001.zip › viruses-1391251-supplementary.pdf]

Table S1. Campania Region Database (CaReDB) characteristics.

Characteristics	
Geographic area	Campania, Italy
Population covered	~5.8 million inhabitants
Age span covered	Whole population
Time span covered a	2010–2019
Scope	Drug utilisation and outcome research; real-world evidences for public health; pharmacoepidemiologic and pharmacoeconomic analysis
Data sources	Specific fields in the data sources contributing to a database
Demographic information	Patient ID; sex; date of birth; municipality of residence; district; local health unit
Outpatient pharmacy records	Patient ID; drug code; prescription date; delivery date; quantity; ATC code; price; defined daily dose; drug distribution channel (file F, file D)
Hospital-discharge records	Patient ID; type of admission; date of admission; reasons for discharge; diagnoses (ICD-9 code); procedures (ICD-9 code); date of discharge; disease-related group
Type of codes for diagnoses	Hospital: ICD-9-CM
Type of codes for medications	ATC classification

ATC = Anatomical Therapeutic Chemical; ICD-9-CM = International Classification of Diseases, 9th Revision, Clinical Modification.
a Time span covered was 2009–2018 for hospital-discharge records and 2014–2019 for outpatient pharmacy records.
